# Comparison of Continuous Thoracic Epidural With Erector Spinae Block for Postoperative Analgesia in Adult Living Donor Hepatectomy

**DOI:** 10.7759/cureus.23151

**Published:** 2022-03-14

**Authors:** Muhammad Zubair, Muhammad Adil Khan, Muhammad Nasir Ayub Khan, Sajida Iqbal, Muhammad Ashraf, Salman A Saleem

**Affiliations:** 1 Pain Management, Shifa International Hospital Islamabad, Islamabad, PAK; 2 Anaesthesiology, Shifa International Hospital Islamabad, Islamabad, PAK; 3 Anaesthesia and Critical Care, Shifa International Hospital Islamabad, Islamabad, PAK; 4 Obstetrics and Gynaecology, Bio Vision Diagnostic Center, Islamabad, PAK

**Keywords:** espb, epidural analgesia, vas, pain management, hepatectomy

## Abstract

Background: Thoracic epidural analgesia (TEA) is commonly used for pain management in donor hepatectomy. Erector spinae plane block (ESPB) is a newer ultrasound-guided block described for the management of thoracic and abdominal pain. There is limited literature available comparing the two techniques. The objective of this study was to compare the postoperative analgesic efficacy and adverse effects of continuous ESPB to continuous TEA in donor hepatectomy.

Methodology: The randomized controlled trial (RCT) was registered on ClinicalTrials.gov (NCT04151511). A total of 82 patients undergoing donor hepatectomy between January 2020 and December 2020 were recruited, of whom 41 received TEA and 41 received ESPB. Randomization was done by the sealed opaque envelope method.

Results: The mean visual analog scale (VAS) scores in donors who received TEA and ESPB in post-anesthesia care unit (PACU) (2.7 + 0.9 vs. 2.4 + 0.5; P = 0.02) at one hour (2.7 + 0.9 vs. 2.2 + 0.6; P = 0.008), six hours (1.8 + 0.9 vs. 0.8 + 0.5; P < 0.001), 12 hours (0.9 + 0.7 vs. 0.2 + 0.7; P < 0.001), and 24 hours (0.48 + 0.5 vs. 0.08 + 0.3; P < 0.001) were significantly different. Mean opioid consumption was 3.38 ± 6.24 mg in the ESPB group and 10.75 ± 9.64 mg in the TEA group (P < 0.001). Mean lung volume (MLV) at 24 hours in the TEA group and ESPB group was 1543 ml and 1815 ml (P < 0.001). MLV was 2545 ml in the TEA group and 2820 ml in the ESPB group at 48 hours (P < 0.001). Mean nausea and vomiting score at six hours was 0.1 vs. 0.03 (P = 0.02).

Conclusion:* *ESPB improves pain control after donor hepatectomy with an enhanced safety profile and reduced opioid consumption.

## Introduction

The occurrence of severe pain following adult living-related donor hepatectomy is 11-37% [1]. Pain is caused by the sub-costal J-shaped incision, the retraction of ribs, and diaphragmatic irritation. Adequate analgesia is important since inadequate pain control can lead to an increased risk of atelectasis, respiratory failure, and prolonged stay in the hospital [2].

Thoracic epidural analgesia (TEA) is frequently used for analgesia in donor hepatectomy. TEA has its own drawbacks, e.g. hypotension, failed analgesia in approximately 20% of patients, intravascular drug delivery, dural puncture, tingling, postoperative coagulopathy, abscess, and an epidural hematoma with resulting paraplegia [3]. A newer regional anesthesia technique is ultrasound-guided erector spinae plane block (ESPB) introduced for thoracic neuropathic pain management in 2016 [4]. It is an avascular plane block associated with fewer procedural complications [5,6]. This block results in lower drug volume in the plasma due to slower absorption of the local anesthetic; hence, the local anesthetic duration of action is prolonged. Various cases have demonstrated its efficacy in various specialties such as acute and chronic pain management, thoracoabdominal surgery, and post-traumatic pain [7,8].

There is limited literature available comparing these two techniques. Therefore, the objective of our study was to compare the postoperative analgesic efficacy and adverse effects of continuous ESPB to that of a continuous TEA in living-related donor hepatectomy surgery. We hypothesized that continuous ESPB would provide superior postoperative analgesia with lesser adverse effects. The primary outcome was postoperative pain scores using the visual analog scale (VAS) for pain at rest in the postoperative period at 0, 1, 6, 12, 24, and 48 hours. The secondary outcomes were the dosage of adjunct opioid used, postoperative nausea and vomiting (PONV), lung volume on incentive spirometry, and hemodynamic parameters in the postoperative period.

## Materials and methods

This study was carried out in accordance with Good Clinical Practice (GCP) guidelines. The trial was registered in November 2019 at ClinicalTrials.gov (NCT04151511). Institutional Review Board and Ethics Committee approved the study protocol on October 19, 2019 (IRB# 165-655-2019). Patient enrolment started after obtaining written informed consent in December 2019.

We included all patients scheduled for elective living donor hepatectomy performed with a right sub-costal J-shaped incision between December 2019 and December 2020. The age range for donors was 18-50 years, with compatible blood group, BMI < 32 kg/m2, related (legally or blood), and in good health. Donors with contraindications to TEA or ESPB, chronic pain conditions, and previous opioid use were excluded.

Standard American Society of Anesthesiologists (ASA) monitors were applied in the operating room. A 20-gauge intravenous cannula was secured in a vein on the upper limb and was connected to a maintenance intravenous fluid. Midazolam 2.5 mg was administered intravenously (IV) for anterograde amnesia and anxiolysis. The patient was preoxygenated at 5 L/min for five minutes using a facemask that was connected to a semi-closed breathing circuit. Buprenorphine 0.3 mg IV was used for surgical analgesia at induction. Propofol (2 mg/kg-1) mixed with lidocaine (60 mg) and atracurium (0.5 mg/kg-1) was administered. Then the endotracheal tube was inserted and controlled ventilation was initiated. A radial arterial catheter and internal jugular central venous catheter (CVC) were placed.

WHO sample size calculator was used for the sample size calculation. Inference for means comparing two independent samples was as follows: value for mu1: 0.41; value for mu2: 0.39; value for sigma: 0.04; value for alpha: 0.10; and value for desired power: 0.80. A total of 37 patients were required in each group. A total of 41 patients were enrolled in each group to compensate for dropouts.

The randomization process was done by a sealed envelope technique. The participants were assigned to either group A, where the patients received continuous TEA, while group B received continuous ESPB. All the blocks were performed by a single experienced anesthesiologist following induction with general anesthesia. Patient and person assessing the patient in the postoperative period were blinded to intervention groups.

The left lateral position was made and a high-frequency linear probe (12 MHz, PLT-1204BT, Toshiba Corp., Tokyo, Japan) in cephalocaudal orientation was used to identify T7-8 or T8-9 interspace over the midline of the back with a Toshiba ultrasound machine (Toshiba Corp., Tokyo, Japan). The area was then prepared and draped using a sterile technique. A sterile 16-gauge Tuohy needle was introduced and advanced using the loss of resistance technique until the epidural space was identified. An epidural catheter of 5 cm length was inserted beyond the depth of skin to space distance. The gravity technique was used to confirm the catheter placement. An initial dose containing 2% lidocaine with 1:200,000 epinephrine (3 mL) was injected to assess any intravascular placement. Transparent sterile dressing was applied.

The left lateral position was made to perform the block. A high-frequency linear probe (12 MHz, PLT-1204BT) in cephalocaudal orientation was used to identify T7-8 or T8-9 interspace over the midline of the back with a Toshiba ultrasound machine. Keeping the probe in the same orientation, it was moved laterally to view the transverse process. The needle was advanced in-plane at 30-45° to hit the transverse process. After negative aspiration, a local anesthetic was injected visualizing the injected spread within the myofascial plane and a catheter was advanced 5 cm. The catheter was secured with a transparent sterile dressing.

General anesthesia was maintained with sevoflurane in a 1:1 combination of oxygen and nitrous oxide. Fluid management with a non-lactated ringer was done using intraarterial blood pressure and central venous pressure monitoring. The patient was extubated after completion of hepatectomy and shifted to the post-anesthesia care unit (PACU).

Postoperative pain was monitored and managed in the PACU and surgical ICU by physicians who were blinded to the study. Continuous infusion of 0.125% bupivacaine through TEA/ESPB catheter was started at 10-12 ml/hour and was titrated to keep the patient's VAS less than five. Paracetamol was used as an adjunct, one gram every six hours. Nalbuphine 5 mg IV was used as an adjunct analgesic if TEA/ESPB was ineffective to provide analgesia or for breakthrough pain. The infusion was stopped on the third postoperative day, VAS was noted in the absence of regional blockade at 72 hours, and the catheter was removed if the VAS score was less than three with the co-analgesics being administered.

Outcomes were assessed based on the VAS, opioid consumption, PONV, and lung volumes. The VAS score and PONV score were assessed at 0, 1, 6, 12, 24, 48, and 72 hours. The total dosage of adjunct nalbuphine used at 24, 48, and 72 hours was also noted. Incentive spirometry lung volumes were noted at 24 and 48 hours. Hemodynamic variations were noted during continuous TEA/ESPB infusions at 0, 1, 6, 12, 24, 48, and 72 hours. Platelet count and international normalized ratio (INR) were noted at 24 and 48 hours.

SPSS version 23.0 (IBM Corp., Armonk, NY) was used to analyze the data. Mean and standard deviations were calculated for the demographic data. Comparative analysis between two groups was done by using an independent sample t-test for the VAS, opioids consumption, the incidence of PONV, and lung volumes. A p-value of <0.05 was considered significant.

## Results

The age range was 18-50 years. The male to female ratio was 27:13 in the TEA group and 25:15 in the ESPB group. Gender distribution between the two groups was statistically insignificant with a p-value of 0.63. Table 1 shows the demographic data comparing the mean age, weight, height, and BMI of the two groups.

**Table 1:** Comparison of demographic data of two groups (n = 40). TEA, thoracic epidural analgesia; ESPB, erector spinae plane block.

	Intervention group	Mean ± SD	P-value
Age (years)	TEA	26.9 ± 7.8	
	ESPB	28.1 ± 8.2	0.5
Weight (kg)	TEA	69.7 ± 7.5	
	ESPB	68.6 ± 8.3	0.5
Height (cm)	TEA	167.8 ± 6.9	
	ESPB	166.4 ± 7.4	0.4
BMI (kg/m2)	TEA	24.6 ± 1.9	
	ESPB	24.6 ± 1.9	0.8

Table 2 shows the comparison of the postoperative nausea vomiting scale at different time intervals between TEA and ESPB groups.

**Table 2:** Comparison of postoperative nausea and vomiting scale at different intervals between two groups using independent samples t-test (n = 40). PONV, postoperative nausea and vomiting; PACU, post-anesthesia care unit; TEA, thoracic epidural analgesia; ESPB, erector spinae plane block.

	Intervention group	Mean ± SD	P-value
PONV scale (0-3) at PACU	TEA	0.70 ± 0.61	
	ESPB	0.18 ± 0.38	<0.001
PONV scale (0-3) at 1 hour	TEA	0.63 ± 0.63	
	ESPB	0.10 ± 0.30	<0.001
PONV scale (0-3) at 6 hours	TEA	0.18 ± 0.38	
	ESPB	0.03 ± 0.16	0.025
PONV scale (0-3) at 12 hours	TEA	0.00 ± 0.00	
	ESPB	0.03 ± 0.16	0.320
PONV scale (0-3) at 24 hours	TEA	0.00 ± 0.00	
	ESPB	0.00 ± 0.00	
PONV scale (0-3) at 48 hours	TEA	0.00 ± 0.00	
	ESPB	0.00 ± 0.00	

Table 3 shows the comparison of postoperative VAS scores at different time points between the two groups using independent samples t-test. A significant difference in VAS score was noted at PACU (P = 0.02), at one hour (P = 0.008), at six hours (P < 0.001), at 12 hours (P < 0.001), and at 24 hours (P = 0.001). The time to first opioid dose in the TEA group was 63.2 versus 132 minutes in the ESPB group. The mean postoperative opioid consumption in the TEA group was 10.7 versus 3.3 (P < 0.001).

**Table 3:** Comparison of postoperative visual analog scale at different intervals between two groups using independent samples t-test (n = 40). VAS, visual analog scale; PACU, post-anesthesia care unit; TEA, thoracic epidural analgesia; ESPB, erector spinae plane block.

	Intervention group	Mean ± SD	P-value
VAS (0-10) at PACU	TEA	2.78 ± 0.92	
	ESPB	2.40 ± 0.54	0.029
VAS (0-10) at 1 hour	TEA	2.75 ± 0.98	
	ESPB	2.25 ± 0.63	0.008
VAS (0-10) at 6 hours	TEA	1.88 ± 0.94	
	ESPB	0.88 ± 0.52	<0.001
VAS (0-10) at 12 hours	TEA	0.98 ± 0.73	
	ESPB	0.28 ± 0.75	<0.001
VAS (0-10) at 24 hours	TEA	0.48 ± 0.59	
	ESPB	0.08 ± 0.35	0.001
VAS (0-10) at 48 hours	TEA	0.05 ± 0.22	
	ESPB	0.08 ± 0.35	0.704

**Figure 1:**
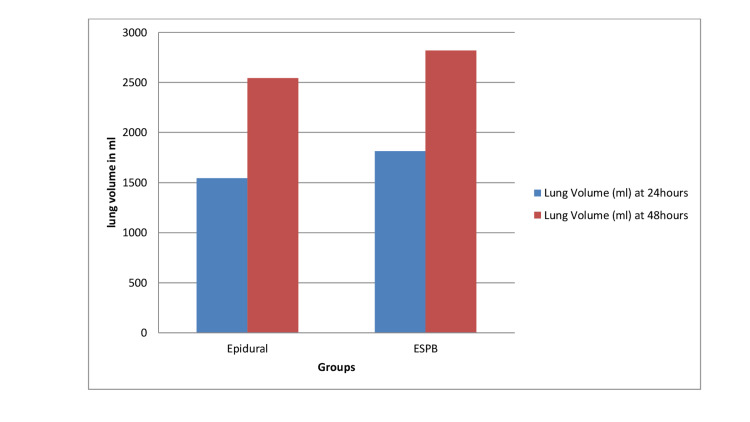
Comparison of postoperative lung volume at different intervals between the two groups. Lung volume was 1543 ml in the thoracic epidural analgesia (TEA) group (n = 40) and 1815 ml in the erector spinae plane block (ESPB) group (n = 40) at 24 hours (P < 0.001). Lung volume was 2545 ml in the TEA group (n = 40) and 2820 ml in the ESPB group (n = 40) (P < 0.001) at 48 hours.

Figure 1 shows lung volume in the TEA group and the ESPB group at 24 and 48 hours.

Hemodynamics parameters like mean arterial pressure and heart rate were comparable between the two groups. The decreasing trend of platelets and the rising trend of INR were noted in both groups.

## Discussion

Regional blocks are an essential part of the multimodal approach for intraoperative and postoperative pain relief in donor hepatectomy. In our study, ESPB provided better analgesia when compared to TEA, as measured by the VAS score and total opioid analgesic consumption. In addition, the incentive spirometry in the ESPB group was superior to the TEA group at 24 and 48 hours. The incidence of PONV was also less in the ESPB group.

The findings of our study are consistent with the findings of a randomized controlled trial for postoperative pain relief after laparoscopic hepatectomy [9]. However, this study differs from the preceding trial in several ways. Firstly, the above-mentioned trial was carried out in laparoscopic hepatectomy where the main component of pain is visceral rather than parietal. Secondly, pain associated with the Thompson retractor is not present in laparoscopic hepatectomy. Finally, a single short ESPB was performed instead of the continuous ESPB used in our study.

The results of this study are similar to the randomized control trials of mini-thoracotomy conducted for pain relief in patients undergoing mitral/tricuspid valve repair, laparoscopic cholecystectomy, open epigastric hernia repairs, and thoracotomies [10,11]. Good postoperative pain control was achieved in studies done on video-assisted thoracic surgery, which is consistent with our study [12,13]. Another study by Wang et al. has the same conclusion as our study, which describes USG ESPB can decrease perioperative opioid consumption and provide better postoperative pain relief in thoracotomy [14].

In our study, patients had higher lung volumes at 24 and 48 hours postoperatively in the ESPB group. Therefore, they did better on spirometry after donor hepatectomy. This was an advantage and might be due to better postoperative pain control and less use of opioids postoperatively. Furthermore, the PONV scores were lower in the ESPB group when compared to the TEA group, which again may be due to less opioid consumption in the ESPB group. A study by Hacibeyoglu et al. also suggests that the ESPB may serve as an innovative, safe, and effective analgesia technique for patients undergoing hepatic surgery, which corresponds to our study findings [15]. Singh et al. also described USG ESPB as effective for postoperative analgesia in choledochal cyst resection surgery, which corresponds to our study findings [16]. The ESPB showed truly effective pain relief and highly variable sensory blockade [17,18].

Both groups experienced a significant decrease in platelet count and an increase in INR following donor hepatectomy. There have been reports of hematoma formation after epidural analgesia in patients with low platelet count and high INR. In our study, however, neither group developed a hematoma. Other authors also report that the ESPB was safe because it targets the fascial plane between the transverse process of vertebras and erector spinae muscles [6,7]. In addition, because the needle path used to perform ESPB targets myofascial plan above the transverse process, it is further away from the spinal cord, pleura, and main vessels. As a result, the risk of hematoma formation and neurological complications is reduced.

Deranged coagulation also puts the patient at risk during removal of the epidural catheter while ESPB catheter can be safely removed even if deranged coagulation is present. This has been described in two studies, which narrate that the ESPB is a safer option in the presence of systemic anticoagulation, use of antiplatelet medications, coagulation disorders, and following heparinization, which are contraindications to TEA [19,20]. Maddineni et al. also described the novel use of the ESPB for analgesia and safe catheter removal in the setting of anticoagulation undergoing major hepatic surgery for intrahepatic cholangiocarcinoma [21]. Therefore, ESPB seems much safer than TEA after donor hepatectomy in the presence of decreased platelets and increased INR.

The limitation of the current study includes a very specific population of healthy voluntary living donors. Generalizability is limited as the study is conducted at a single tertiary care facility. Another limitation is the experience required to perform ESPB. In the current study, all blocks were performed by senior anesthesiologists. The replication of these results by physicians of variable experience remains to be determined.

## Conclusions

ESPB appears to be a superior method of regional block for postoperative analgesia when compared with TEA. It reduces the frequency of nausea and vomiting, decreases opioid use, and improves lung volumes when compared with TEA. Hypotension was not observed in both groups but ESPB avoids the theoretical risk of the sympathetic block. In hepatectomy, deranged coagulation is one of the factors that restrict the epidural catheter removal but ESPB catheter can be removed even with deranged coagulation. The findings of the current study need to be confirmed in clinical practice in other centers to validate these results.
